# Efficacy and safety of transcranial direct current stimulation (tDCS) in treatment of refractory epilepsy: an updated systematic review and meta-analysis of randomized sham-controlled trials

**DOI:** 10.1007/s10072-024-07866-1

**Published:** 2024-11-13

**Authors:** Yousef Hawas, Abdallah Abbas, Ibraheem M. Alkhawaldeh, Mohamed Abo Zeid, Mohammad Al Diab Al Azzawi, Hamza Khaled Alsalhi, Ahmed Negida

**Affiliations:** 1 Faculty of Medicine, Tanta University, Gharbeya, Egypt; 2 Faculty of Medicine, Al-Azhar University, Damietta, Egypt; 3 Faculty of Medicine, Mutah University, Al-Karak, Jordan; 4 Faculty of Medicine, The National Ribat University, Khartoum, Sudan; 5 Faculty of Medicine, The Hashemite University, Zarqa, Jordan; 6 Department of Neurology, Virginia Commonwealth University, Richmond, Virginia USA; 7 Medical Research Group of Egypt, Negida Academy, Arlington, MA USA

**Keywords:** Transcranial direct current stimulation, Refractory epilepsy, Seizure frequency, Interictal epileptiform discharges

## Abstract

Despite the currently available treatment, one-third of epilepsy patients continue to experience seizures. Transcranial direct current stimulation (tDCS) has emerged as a potential neuromodulation approach for the non-invasive treatment of refractory epilepsy. This study aims to provide a comprehensive investigation of the efficacy and safety of tDCS in patients with drug-resistant epilepsy. The following databases were searched from inception until June 2023; PubMed, Scopus, Embase, WOS, EBSCO, Cochrane Central, and Ovid MEDLINE. Pooled mean difference was calculated for change in seizure frequency (SF), and number of Interictal epileptiform discharges (IEDs) at different follow-up intervals. We included nine parallel randomized sham-controlled trials with a total of 267 patients. Active tDCS patients had a significantly lower SF per month at 4 and 8 weeks (MD = -4.06, 95% CI [-6.01 to -2.12], *p* < 0.0001), and (MD = -2.66, 95% CI [-5.09 to -0.23], *p* = 0.03), respectively. However, weekly SF showed no statistically significant results at 4 weeks of follow-up. The IEDs were observed to significantly decline at 2, 4, and 8 weeks of follow-up. The reported adverse events were mild including mild itching and erythematous rash that resolved spontaneously. In conclusion, tDCS significantly reduced monthly SF and the number of IEDs. Future large RCTs with standard clear informed parameters are still required.

**Supplementary Information:**

The online version contains supplementary material available at 10.1007/s10072-024-07866-1.

## Introduction

Approximately 50 million people, which is 1% of the global population, are affected by epilepsy. According to the International League Against Epilepsy (ILAE), drug-resistant epilepsy occurs when a patient does not respond adequately to at least two properly chosen anti-seizure medications (ASMs) at the appropriate dosage [1]. The prevalence of refractory epilepsy reached 13.7% and 36.3% in community-based studies and clinic-based cohort studies, respectively [2]. Temporal lobe epilepsy, which accounts for 30–40% of cases, is the most prevalent form of refractory epilepsy [3].

Various approaches, such as neurosurgery methods [4], the ketogenic diet [5], and neurostimulation, have been proposed as treatment choices. Patients with refractory epilepsy should be assessed for possible surgery [6]. Nonetheless, not all patients are suitable candidates for surgery due to the location of the epileptogenic zone in the eloquent cortex, lengthy waiting lists, unavailability in numerous regions of the world, and the potential for seizures to persist even after surgery, which diminishes the appeal of surgical intervention for many patients [7].

Neurostimulation involves using electrical impulses to activate the brain to reduce the frequency and severity of seizures, making it a viable option for patients who are not suitable candidates for epilepsy surgery [8]. Furthermore, it shows potential as a treatment for refractory epilepsy in pediatrics. However, there is no established standard for choosing brain stimulation therapy for children with refractory epilepsy. Currently, treatment decisions are tailored to each patient’s circumstances. The only neuromodulation therapies approved by the FDA for the management of refractory epilepsy in adults are vagus nerve stimulation (VNS), deep brain stimulation (DBS), and responsive neurostimulation (RNS) [9].

Transcranial direct current stimulation (tDCS) is a long-standing technique for cortical stimulation that can non-invasively alter neuronal excitability in humans [10]. Its safety, ease of use, and widespread availability have made it popular in neuropsychiatry. It has been trialed on conditions such as ADHD, schizophrenia, cerebral palsy, dystonia, and stroke [11–18]. This technique involves applying a low-intensity current (1–2 mA) that can influence the membrane potentials in two different ways. Cathodal tDCS (c-tDCS) causes hyperpolarization of the resting membrane potentials, while anodal tDCS causes depolarization [10]. The potential to modify seizure network excitability using tDCS offers a noninvasive option for decreasing the abnormal networks excitability in epilepsy patients, potentially leading to a reduction in rates of seizure in this population [10]. High-definition-tDCS (HD-tDCS) is preferred over conventional tDCS because it allows for more precise and focused delivery of electrical stimulation to targeted brain regions. By using smaller electrodes, the technique enhances the focality of the stimulation, limiting neuromodulatory effects on adjacent areas. However, the increased current density with smaller electrode surfaces raises the risk of burns, requiring the application of a lower current for safety. Despite the reduced current, the focused delivery enables a higher intensity of stimulation to reach the cortex [19].

tDCS has several advantages over DBS and VNS. Unlike DBS and VNS, tDCS is non-invasive, avoiding surgical risks such as infection and inflammation. It is generally safe and well-tolerated, with minor side effects like tingling or itching under the electrodes [20]. tDCS is also more cost-effective, with simpler and more portable equipment, making it accessible for clinical and research use [20]. The ease of use and portability of tDCS contrasts with the complex surgical procedures required for DBS and VNS. tDCS allows for better experimental control through indistinguishable sham (placebo) stimulation, facilitating double-blind studies and reducing bias [20]. Additionally, tDCS can be easily combined with pharmacotherapy, enhancing therapeutic outcomes [20]. Its ability to target specific brain regions by adjusting electrode placement makes it versatile for various applications.

This systematic review and meta-analysis aim to comprehensively evaluate the efficacy of tDCS in reducing seizure frequency (SF) and interictal epileptiform discharges (IEDs) in children and adults with drug-resistant epilepsy.

## Methods

This systematic review and meta-analysis was conducted following the Preferred Reporting Items for Systematic Reviews and Meta-Analyses (PRISMA) statement [21]. The Cochrane Handbook guidelines were followed in conducting all the steps [22].

### Literature search

We searched PubMed, Embase, EBSCO, Web of Science, Cochrane Central, Ovid Medline, and Scopus. Studies from inception to June 2023 were retrieved using the following search strategy “((tDCS OR Transcranial direct current stimulation) AND (Pediatric Epilepsy OR Focal Epilepsy OR Refractory Epilepsy OR Epilepsy))”.

We included studies with the following PICO criteria: Population (P): Adults or/and Children with any type of drug-resistant epilepsy.; Intervention (I): Active tDCS; Control (C): Sham tDCS; Outcomes (O): Frequency of Seizures (SF), Interictal Epileptiform Discharges (IEDs), and side effects.; Study design (S): Parallel randomized sham-controlled trials. The exclusion criteria were as follows: non-English studies, case reports, animal studies, reviews, editorials, studies with only an abstract or unavailable full text or overlapped data, and studies with other interventions or without a comparison group. Cross-over RCTs were excluded (Online Resource 1).

### Study selection

The literature search results were collected in an online Excel sheet and screened by two independent authors. Titles and abstracts of retrieved studies were screened, and then the screening of the full text of eligible articles. Any conflicts resolved by consensus.

### Data extraction

Two authors (Y.H and H.K.A) independently extracted the data in prepared Excel sheets. The extracted data from the included studies were the summary, baseline characteristics, and outcomes. The summary of the included studies involved the study design, country, number of participants, number of centers, refractory epilepsy type and etiology, tDCS electrodes, cathode and anode position, electrode size, current, and density, number of sessions, duration of follow-up, primary outcomes, ameliorates, and adverse events. The baseline characteristics of patients included age during the study, age at onset of seizures, sex, number of seizures per day, per week, and per month, and IEDs per 30 min. The primary outcomes were: (1) SF per month at four weeks and eight weeks, and per week at four weeks of follow-up. (2) IEDs per 30 min at two, four, and eight weeks of follow-up. The secondary outcome was the adverse events. The baseline and outcome data are characterized by the varied presentations between the studies. In, Sanjuan et al. study [23], data were provided for each individual participant, with post-intervention results of the SF expressed as a percentage change. Hence, to calculate the exact post-intervention value the following formula was applied:

$$\begin{gathered}\:Post\:intervention\:value = \hfill \\\,\,\,\,\,\,\:\frac{{Percent\:change\:value\: \times \:\:Baseline\:value}}{{100\% }}\: \hfill \\\,\,\,\,\,\, + Baseline\:value \hfill \\ \end{gathered}$$

Then the data for each group were calculated and compiled into mean and SD for post- and pre-intervention values using the Meta-Analysis Accelerator tool [24]. After that, the mean change for each group was calculated using the same tool [24]. In other studies, including Yang et al. 2019 [25], Auvichayapat et al. 2013 [17], and Auvichayapat et al. 2016 [26], the data were not clearly described in the text and were presented in figures. Therefore, the data were extracted from the presented figures using Web plot digitizer version 4.7, which is an advanced computer vision-powered software that facilitates the extraction of continuous data from a wide range of graphical data visualizations [27]. The data could not be extracted from Luo et al. 2021 as it was normalized to 100% [28].

### Quality assessment

For the assessment of the included studies, two authors independently evaluated them. If there was a disagreement, it was resolved by consensus. We assessed the quality of the included randomized clinical trials using the Cochrane tool risk of bias ROB-II [29]. The tool involves five major domains: the assessment of the risk of bias in the randomization process, the risk of deviation from intended interventions, the risk of missing outcome data, the risk of bias in the measurement of the outcomes, and the risk of selecting the reported results. Each domain was judged as either low risk, with some concerns, or a high risk of bias.

### Statistical analysis

We performed the analysis using the online RevMan web. It is a widely utilized software for conducting systematic reviews and meta-analyses, endorsed by the Cochrane Collaboration. In addition to its user-friendly interface, it facilitates the creation of forest plots. The transition to the online version, RevMan Web, has further enhanced accessibility, allowing users to work from any internet-enabled device without the need for installation [30, 31]. The mean difference (MD) and its 95% confidence interval (CI) were calculated for continuous outcomes. The mean change for all included studies was calculated using the Meta-Analysis Accelerator tool [24]. Heterogeneity was evaluated using the Chi-square and I-square tests. Heterogeneity was considered significant if the p-value was below 0.1, while the Isquare test results were interpreted as substantial heterogeneity for 50–90%, moderate heterogeneity for 30–60%, and not significant for 0–40% [32].

## Results

### Literature search results

**Fig. 1:**
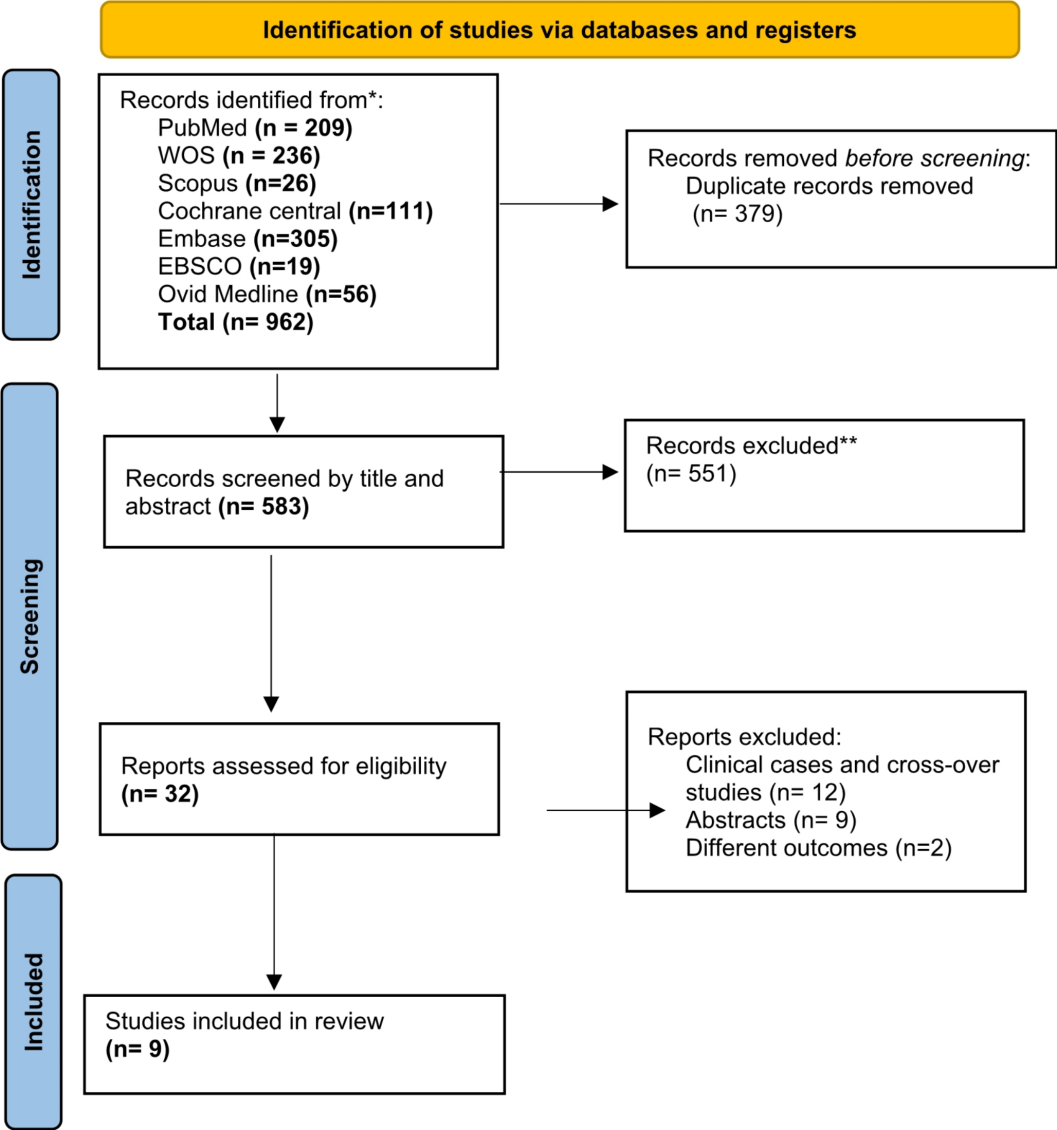
PRISMA 2020 flow diagram for new systematic reviews which included searches of databases and registers only

Our systematic search yielded 962 potential studies. After eliminating 379 duplicates, a further exclusion of 551 studies occurred during the title and abstract screening. As a result, a total of 32 articles went full-text screening resulting in the exclusion of an additional 23 studies. Ultimately, nine studies met the eligibility criteria for inclusion in our review **(**Fig. 1**)**.

### Characteristics of included studies

All the studies incorporated into our analysis were parallel randomized sham-controlled trials, comprising a total of 267 patients with their mean age ranging from 6.29 to 38 years. Two studies were conducted on children below 18 years of age. Six studies included adults at or above 18 years of age. Only one study (Fregni et al. 2006) included mixed participants below and above 18 years of age. Two RCTs were conducted in Iran, two in Thailand, two in China, and three in Mexico, Australia, and Brazil. Notably, all RCTs applied conventional tDCS electrodes except Rezakhani et al. who applied HD-tDCS electrodes. The number and duration of sessions varied throughout the trials ranging from one session for 18 min up to 14 sessions each for 20 min. Demographic distribution revealed that 61.04% of the study participants were males (Table 1). The baseline characteristics of participants are shown in Table 2.

**Table 1:** General characteristics of the included studies

Study ID	Study design	Number of participants	Country	Number of centers	Refractory epilepsy type	tDCS electrodes	Cathode position	Anode position	Electrode size, current, and density	Number and duration of sessions	Follow-up durations	Primary outcomes	Ameliorates	Adverse events
***Ashrafzadeh 2023***	**Parallel RCT**	**18 children**	**Iran**	**Single**	**Focal**	**Conventional tDCS**	**EEG 10–20 (epileptogenic focus)**	**Contralateral deltoid muscle**	**0.15 cm2**,** 1–2 mA**,** 66.7–133.3 A/m2**	**5 sessions each for 20 min**	**at 4 weeks**	**EEG**,** EDs**,** SF**,** Seizure duration**	**No statistically significant difference between active and sham groups in terms of EDs and SF. However**,** tDCS significantly reduced seizure duration in the active group.**	**3 patients with mild itching that was easily tolerated**
***Rezakhani 2022***	**Parallel RCT**	**20 Adults**	**Iran**	**Single**	**Focal**	**High-Definition tDCS**	**EEG 10–20 (epileptogenic focus)**	**Contralateral head**	**3.14 cm2**,** 2 mA**,** 6.369 A/m2**	**10 sessions over 2 weeks (5 consecutive sessions each week) each for 30 min**	**at 2**,** 4**,** 8**,** and 12 weeks**	**EEG (32 electrodes)**,** EDs**,** SF**	**cathodal HD-tDCS showed a significant reduction in SF and EDs**	**The patients experienced initial itching and tingling sensation.**
***Luo 2021***	**Parallel RCT**	**25 Adults**	**China**	**Single**	**Focal**	**Conventional tDCS**	**EEG 10–10 (epileptogenic focus)**	**contralateral supraorbital or post-temporal**	**1 mA**	**5 sessions each for 20 min**	**Immediately**,** and at 4 weeks**	**EEG (16 electrodes)**,** EDs**,** SF**	**Decrease the IEDs by 30.2% immediately and 33.4% at 4 weeks of the active group compared to baseline. ctDCS ameliorated the EEG functional network. the small-worldness index significantly reduced by 3.5% compared to the baseline. No obvious change in seizure frequency during follow-up.**	**one patient in the active group reported dizziness that was relieved within 24 h.**
***Yang 2020***	**Parallel RCT**	**70 Adults**	**China**	**Four**	**Focal**	**Conventional tDCS**	**EEG 10–20 (epileptogenic focus)**	**Contralateral head**	**11.9 cm2**,** 2 mA**,** 1.7 A/m2**	**14 sessions each for 20 min (1 × 20 min) or 40 min (2 × 20 min with 20 min interval (20-20-20))**	**The first 8 weeks**	**SF**	**Compared to the sham group**,** the (1 × 20 min) group showed a significant − 50.73 − 21.91% reduction in SFs that lasted for 4 weeks. (2 × 40 min) group showed a significant − 63.19 − 49.79% reduction in SFs that lasted for 5 weeks. (2 × 40 min) the group had a − 64.98 − 66.32% greater reduction in SFs when compared to (1 × 20 min) group**	**19 patients in the (1 × 20 min) group**,** 21 in the (2 × 20 min) group**,** and 2 in the sham group reported mild itching sensation. 3 patients in the (2 × 20 min) group and 2 in the sham group experienced a focal onset impaired awareness seizure during the session.**
***San juan 2016***	**Parallel RCT**	**28 Adults**	**Mexico**	**Single**	**MTLE-HS**	**Conventional tDCS**	**EEG 10–20 (epileptogenic focus)**	**Contralateral supraorbital**	**35 cm2**,** 2 mA**,** 0.571 A/m2**	**3 or 5 sessions for 30 min**	**Immediately**,** 1**,** 2 months**	**EEG**,** EDs**,** SF**	**Reduction of SF immediately**,** 1 and 2 months post-tDCS (3 days: −43.4%; 5 days: −54.6%) compared to the sham group − 6.25%.**	**Mild headache and itching**
***Zoghi 2016***	**Parallel RCT**	**29 Adults**	**Australia**	**Single**	**Focal TLE**	**Conventional tDCS**	**Affected temporal lobe**	**Contralateral supraorbital**	**12 cm2**,** 1 mA**,** 0.833 A/m2**	**1 session for 18 min with 20 min interval (9-20-9)**	**at 4 weeks**	**EEG**,** Paired-pulse transcranial magnetic stimulation: SICI calculated from MEPs**,** SF**	**Increase in SICI for the active group compared to sham**,** Decrease of SF by 42% in the active group**,** and reduced seizure duration.**	**itching**,** tingling**,** burning sensation; headache**,** and neck pain**,
***Auvichayabat 2016***	**Parallel RCT**	**22 children**	**Thailand**	**Single**	**Lennox-Gastaut syndrome**	**Conventional tDCS**	**EEG 10–20 (C3)**	**Contralateral right shoulder**	**35 cm2**,** 2 mA**,** 0.571 A/m2**	**5 sessions each for 30 min**	**at 1**,**2**,**3**,** and 6 weeks**	**EEG (32 electrodes)**,** EDs**,** SF**,** and O2 sat Monitoring**	**Five consecutive days of cathodal tDCS over M1 combined with pharmacologic treatment appears to decrease SF by 89.75% up to 1 month and IED by 76.48% up to 3 months that lasted for 3 weeks.**	**1 patient with mild itching that relieved spontaneously**
***Auvichayabat 2013***	**Parallel RCT**	**36 children**	**Thailand**	**Single**	**Focal**	**Conventional tDCS**	**EEG 10–20 (epileptogenic focus)**	**Contralateral shoulder**	**35 cm2**,** 1 mA**,** 0.285 A/m2**	**1 session for 20 min**	**Immediately**,** 24 h**,** 48 h and 4 weeks**	**EEG (32 electrodes)**,** EDs**,** SF**	**Decrease of EDs by 45.3% at immediate**,** 24 h**,** and 48 h**,** however**,** it increased at 4 weeks. a small reduction in SF (4.8%) at 4 weeks**	**1 patient with transient skin erythema**
***Fregni 2006***	**Parallel RCT**	**19 Adolescents and adults**	**Brazil**	**Single**	**Focal and multifocal**	**Conventional tDCS**	**EEG 10–20 (epileptogenic focus in focal**,** and Cz in multifocal)**	**Contralateral head or supraorbital**	**35 cm2**,** 1 mA**,** 0.285 A/m2**	**1 session for 20 min**	**Immediately**,** 15 days**,** 30 days**	**EEG (21 electrodes)**,** EDs**,** SF**	**significant reduction in the number of EDs by − 64.3% and SF by − 44% in the active group**	**Itching sensation in 3 patients of the active group**,** and 1 patient in the sham group**

***EDs***: *Epileptiform Discharges*, ***SF***: *Seizure Frequency*, ***MTLE-HS***: *Mesial Temporal lobe epilepsy with hippocampal sclerosis*, ***TLE***: *Temporal lobe epilepsy and****SICI***: *short interval intracortical inhibition*

**Table 2:** Baseline characteristics of the participants

Study ID	Age (Years) (Mean ± SD)		Age at onset of seizures (years) (Mean ± SD)		Sex (Male) *N*. (%)		Number of patients		Seizure frequency per days (Mean ± SD)		Seizure frequency per week (Mean ± SD)		Seizure frequency per month (Mean ± SD)		Interictal discharge frequency per 30 min (Mean ± SD)		Duration of seizure (Mean ± SD)	
	Intervention	Comparator	Intervention	Comparator	Intervention	Comparator	Intervention	Comparator	Intervention	Comparator	Intervention	Comparator	Intervention	Comparator	Intervention	Comparator	Intervention	Comparator
***Ashrafzadeh 2023***	**9.67 ± 3.08**	**9.22 ± 3.89**	**NR**	**NR**	**8 (88.8%)**	**6 (66.7%)**	**9**	**9**	**NR**	**NR**	**12.89 ± 19.38**	**16.78 ± 11.3**	**NR**	**NR**	**54.22 ± 29.05**	**35.1 ± 17.07**	**24.33 ± 17**	**27.22 ± 20**
***Rezakhani 2022***	**26.5 ± 6.5**	**29.3 ± 9.19**	**15.8 ± 5.2**	**18.2 ± 5.39**	**6 (60%)**	**5 (50%)**	**10**	**10**	**NR**	**NR**	**NR**	**NR**	**8.20 ± 2.39**	**8.40 ± 2.27**	**10.10 ± 2.38**	**10.00 ± 2.31**	**NR**	**NR**
***Yang 2020 1 × 20 min***	**30.58 ± 10.45**	**30.19 ± 13.03**	**NR**	**NR**	**12 (50%)**	**12 (57.14%)**	**24**	**21**	**NR**	**NR**	**4.97 (1.221)**	**4.02 (1.53)**	**NR**	**NR**	**NR**	**NR**	**NR**	**NR**
***Yang 2020 2 × 20 min***	**31.8 ± 9.25**	**30.19 ± 13.03**	**NR**	**NR**	**18 (72%)**	**12 (57.14%)**	**25**	**21**	**NR**	**NR**	**3.84 (0.83)**	**4.02 (1.53)**	**NR**	**NR**	**NR**	**NR**	**NR**	**NR**
San juan 2016 30Min*3d	**NR**	**NR**	**NR**	**NR**	**6 (50)**	**6 (75%)**	**12**	**8**	**NR**	**NR**	**NR**	**NR**	**7.167 ± 3.41**	**11.375 ± 9.41**	**NR**	**NR**	**32**	**21.1**
San juan 2016 30Min*5d	**NR**	**NR**	**NR**	**NR**	**4 (50%)**	**6 (75%)**	**8**	**8**	**NR**	**NR**	**NR**	**NR**	**9.125 ± 8.92**	**11.375 ± 9.41**	**NR**	**NR**	**21**	**21.1**
***zoghi 2016***	**38 ± 13**		**NR**	**NR**	**11 (37.9%)**		**20**	**9**	**NR**	**NR**	**NR**	**NR**	**53 ± 78.95**	**20.28 ± 33.69**	**NR**	**NR**	**NR**	**NR**
***Auvichayabat 2016***	**6.67 ± 1.54**	**6.29 ± 1.98**	**2.32 ± 2.39**	**1.58 ± 1.70**	**9 (60%)**	**5 (71.4%)**	**15**	**7**	**80.67 ± 54.43**	**93.43 ± 59.94**	**NR**	**NR**	**NR**	**NR**	**640.13 ± 263.30**	**690.31 ± 307.33**	**NR**	**NR**
***Auvichayabat 2013***	**11.80 ± 2.1**	**11.12 ± 1.61**	**7.3 ± 1.2**	**6.8 ± 2.3**	**20 (74.1%)**	**6 (66.7%)**	**27**	**9**	**NR**	**NR**	**NR**	**NR**	**11.67 ± 4.63**	**10.22 ± 1.72**	**538.44 ± 279.25**	**582 ± 244.19**	**NR**	**NR**
***Fregni 2006***	**24.0 ± 9.8**	**24.3 ± 6.4**	**NR**	**NR**	**5 (50%)**	**6 (66%)**	**10**	**9**	**NR**	**NR**	**NR**	**NR**	**7.2 ± 3.6**	**8.4 ± 5.6**	**NR**	**NR**	**NR**	**NR**

### Quality assessment

**Fig. 2:**
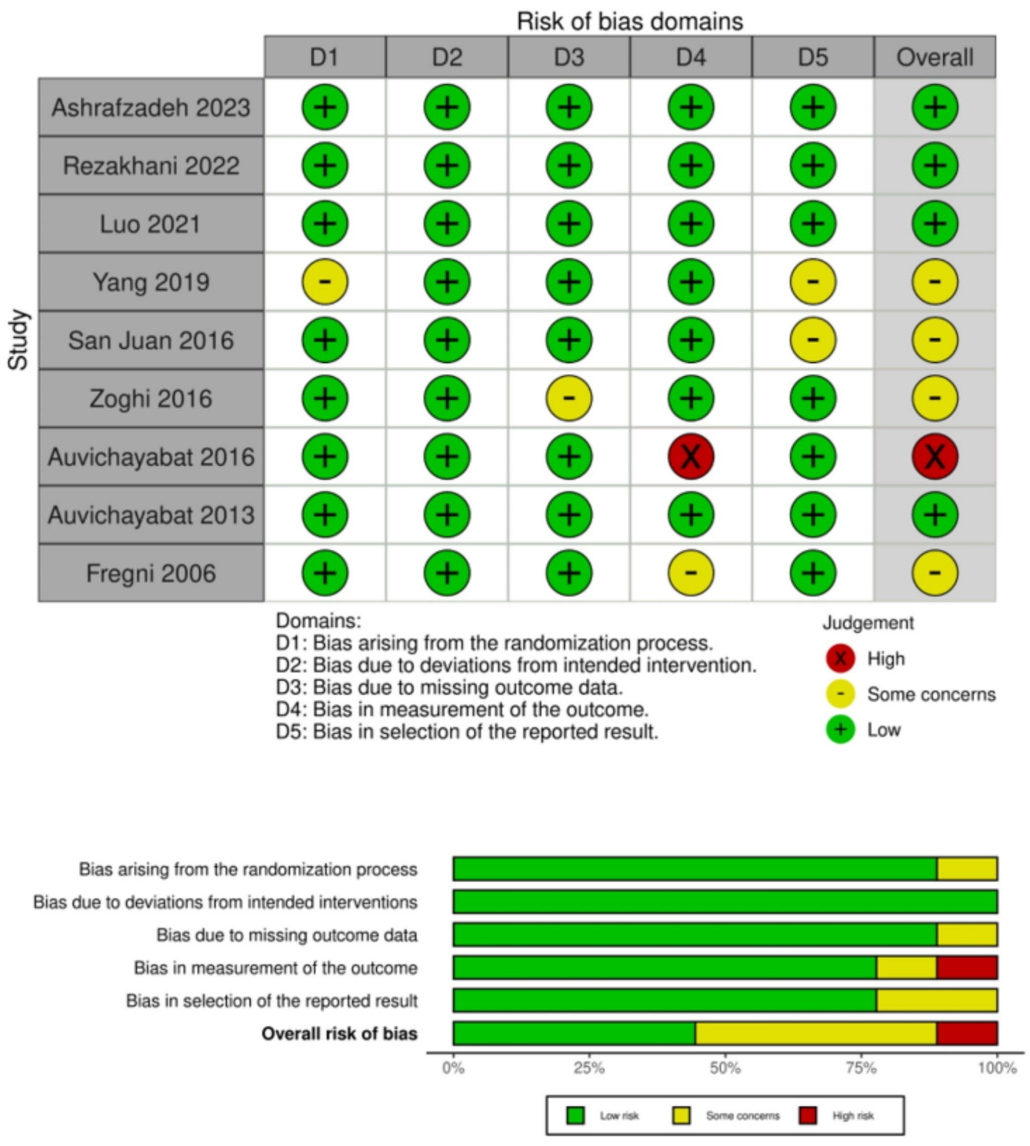
Quality assessment of the included studies

Using the Cochrane RoB2 tool’s five domains, we evaluated each outcome included in the quantitative synthesis’s risk of bias. Of the nine studies, four indicated a low risk of bias, four studies exhibited some concerns, and one showed a high risk (Fig. 2).

### Outcomes

#### Interictal epileptiform discharge rate

We evaluated the interictal epileptiform discharge rate at different points of follow-up to estimate the impact of tDCS in the long run. As a result, our analysis showed statistically significant results favoring active tDCS over sham tDCS at two, four, and eight weeks of follow-up.

**Fig. 3:**
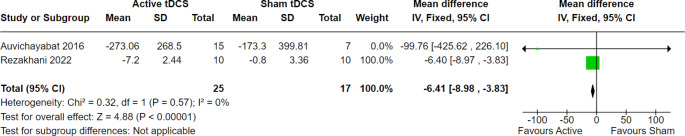
Interictal epileptiform discharges at 2 weeks of follow-up

**Fig. 4:**
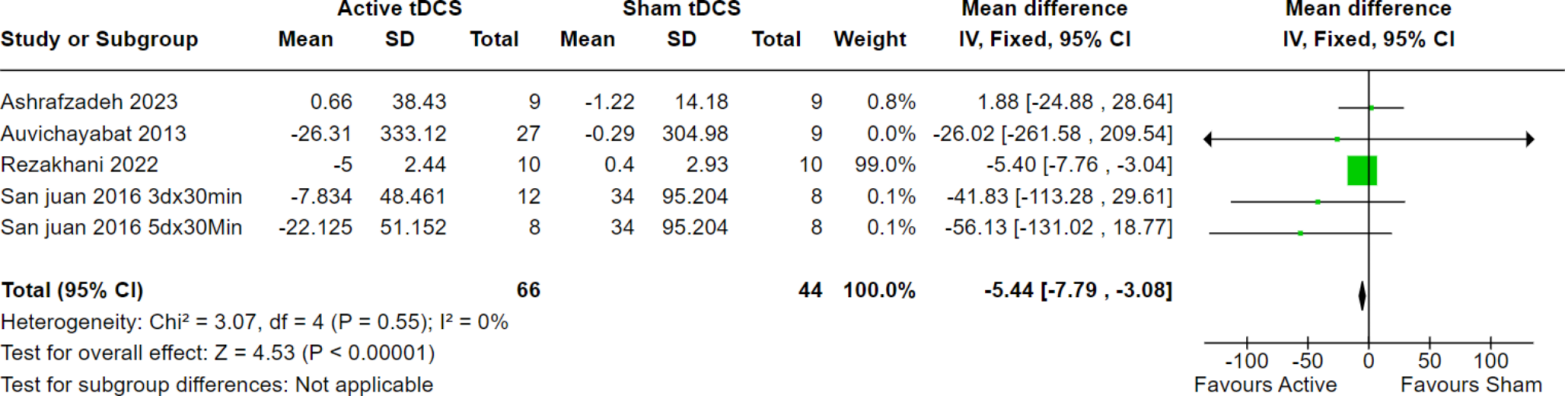
Interictal epileptiform discharges at 4 weeks of follow-up

**Fig. 5:**
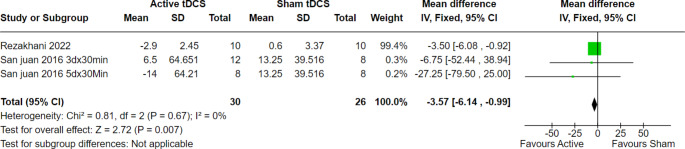
Interictal epileptiform discharges at 8 weeks of follow-up

At two weeks of follow-up, 42 patients from two trials were evaluated, and the overall effect estimate showed significant results favoring active tDCS over the sham (MD = -6.14, 95% CI [-8.98 to -3.83], *p* < 0.00001), with no heterogeneity among the included studies (*p* = 0.57, I2 = 0%) (Fig. 3). Furthermore, at four weeks of follow-up, 110 patients from five trials were evaluated, and the overall effect estimate showed significant results favoring active tDCS over the sham procedure (MD = -5.44, 95% CI [-7.79 to -3.08], *p* < 0.00001), with homogeneity among the included trials (*p* = 0.55, I2 = 0%) (Fig. 4). Finally, at eight weeks of follow-up, 56 patients from three trials were evaluated, and the overall effect estimate showed significant results favoring active tDCS over the sham procedure (MD = -3.57, 95% CI [-6.14 to -0.99], *p* = 0.007), with homogeneity among the included trials (*p* = 0.67, I2 = 0%) (Fig. 5).

#### Seizure frequency per month

SF was assessed at four and eight weeks of follow-up per month and it demonstrated statistically significant results at each point.

**Fig. 6:**
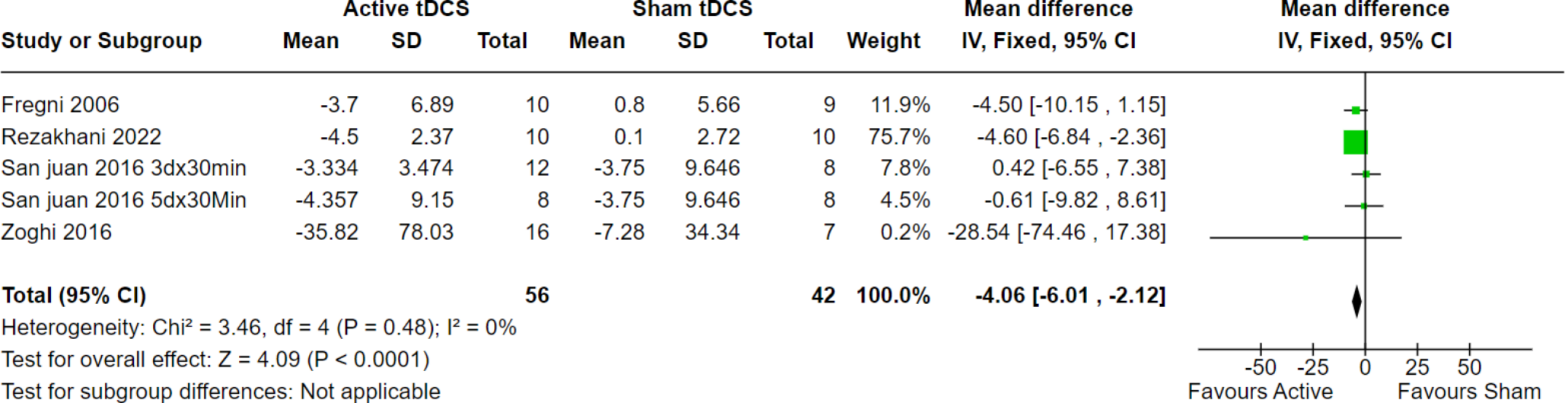
Seizure frequency per month at 4 weeks of follow-up

**Fig. 7:**
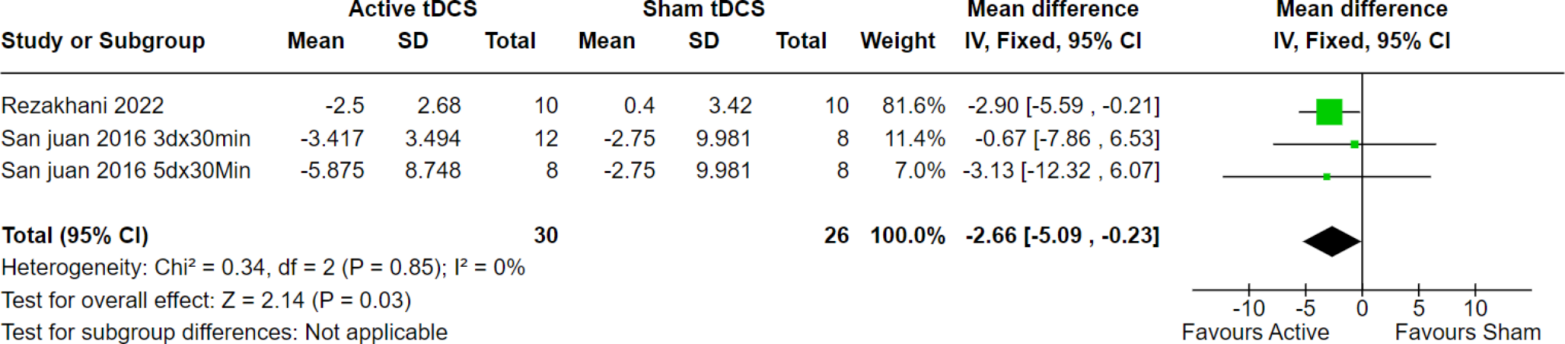
Seizure frequency per month at 8 weeks of follow-up

At four weeks of follow-up, 98 patients across five trials were evaluated, and the overall effect estimate showed significant results favoring active tDCS over the sham technique (MD = -4.06, 95% CI [-6.01 to -2.12], *p* < 0.0001), with no heterogeneity among the included studies (*p* = 0.48, I2 = 0%) (Fig. 6). Moreover, at eight weeks of follow-up, 56 patients from three trials were evaluated, and the overall effect estimate showed significant results favoring active tDCS over the sham procedure (MD = -2.66, 95% CI [-5.09 to -0.23], *p* = 0.03), with homogeneity among the included trials (*p* = 0.85, I2 = 0%) (Fig. 7).

#### Seizure frequency per week

**Fig. 8:**
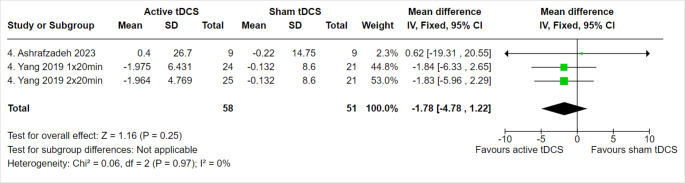
Seizure frequency per week at 4 weeks of follow-up

We evaluated SF per week at four weeks of follow-up. As a result, 109 patients from three trials were evaluated. The overall effect estimate did not favor either active tDCS or sham tDCS at four weeks of follow-up (MD = -1.78, 95% CI [-4.78 to 1.22, *p* = 0.25), with no heterogeneity among the included trials (Fig. 8). Further analyses for SF sub-grouped based on intensity, age, and follow-up periods are in supplementary files (Online Resource 2).

## Discussion

We found that tDCS significantly reduces IEDs and SF in patients with drug-resistant epilepsy. Active tDCS was more effective than sham tDCS in reducing IEDs at two, four, and eight weeks of follow-up, with consistent and statistically significant improvements and no observed heterogeneity among the studies.

Additionally, active tDCS significantly reduced monthly SF. At four and eight weeks of follow-up, the reduction in monthly SF was significant and consistent across studies, with no observed heterogeneity. Weekly SF showed no significant reduction at four weeks of follow-up. Overall, these findings suggest that tDCS may be an effective intervention for reducing seizure activity in individuals with drug-resistant epilepsy. The therapeutic effects of tDCS in epilepsy involve various neurophysiological processes.

Neuronal membranes are hyperpolarized by c-tDCS, resulting in an increased threshold for action potentials and suppression of spontaneous excitatory postsynaptic currents [26, 33]. This process leads to long-term depression (LTD) of synapses, which depends on N-methyl-D-aspartate (NMDA) receptors and metabotropic glutamate receptors, ultimately reducing cortical excitability and producing anticonvulsant effects [26, 33].

Moreover, tDCS alters membrane potential and synaptic efficacy, promoting inhibitory connections and impeding excitatory ones. This process selectively diminishes cortical excitability and decreases seizure frequencies [23, 28]. Cathodal stimulation has the potential to reduce glutamatergic activity and elevate GABA levels, thereby tipping the balance toward inhibition. These changes are due to both electrolytic and synaptic modifications, which play a critical role [34].

Furthermore, tDCS influences the dynamics of brain networks by reducing synchronization levels in epileptic focal points, which is associated with a decrease in seizures [28]. It is believed that the persistent effects of tDCS result from changes in the efficacy of NMDA receptors, leading to a decrease in presynaptic input and a reduction in synaptic strength [25, 28]. Overall, these mechanisms collectively inhibit hyperexcitability and modulate brain activity, making tDCS a promising noninvasive treatment for drug-resistant epilepsy.

Most of the included studies demonstrated a significant impact on SF and IEDs. However, Ashrafzadeh et al. (2023) found no significant difference in the SF or the number of spikes and sharp waves in children but observed a significant reduction in the duration of seizures in the active tDCS group [35]. While, in children too, Auvichayapat et al. (2016) and (2013) found significant reductions in IEDs and SF in the tDCS group compared to the sham group [26, 36].

In addition, Fregni et al. (2006) reported a significant reduction in IEDs after c-tDCS treatment, with a 76.48% decrease immediately after stimulation that persisted at one-, two-, and three-week follow-ups, although the effect diminished over time and was not significant at the four-week follow-up compared to the sham group. Also, they observed a trend toward a decrease in SF, though it was not statistically significant [33]. All studies applied the c-tDCS except Rezkhani et al. 2022 who used the HD-tDCS. The effect of HD-tDCS resulted in a significant reduction of IEDs and SF that was maintained for over 12, and eight weeks respectively in patients with refractory focal epilepsy in Rezakhani et al. (2022) [37]. Recent trials employed HD-tDCS in patients with refractory status epilepticus and have shown promising results with no adverse events [38–40].

Zoghi et al. (2016) observed a significant reduction in IEDs in the active group compared to the sham group, with significant changes maintained for up to three weeks post-treatment. They found a 42% reduction in SF after a single session for 18 min with 20 min interval (9-20-9) of c-tDCS [41]. Yang et al. (2019) observed a significant reduction in SF after tDCS treatment, especially in the group that received two sessions each for 20 min per day than those who received a 20-minute session at two, four, six, and eight weeks of follow-up [25].

Furthermore, San-Juan et al. (2016) demonstrated a significant reduction in SF in both three- and five- days c-tDCS groups compared to the sham group at two months follow-up. There was no significant difference in IEDs at any of the follow-up intervals except immediately post-tDCS which was slightly significant compared to baseline [23]. In addition, Luo et al. (2021) demonstrated a significant reduction in IEDs in the active c-tDCS group, with a 30.2% reduction after five days of treatment that became statistically significant at the four-week follow-up with a 33.4% reduction. However, they reported less consistent reductions in SF despite significant reductions in IEDs [28].

All participants in the included studies continued using ASMs alongside tDCS, except for Zoghi et al.2016, who did not specify whether ASMs were used. Additionally, none of the included trials detailed the specific types of ASMs used. This highlights the need for further investigation into whether the concurrent use of ASMs could potentially overestimate or underestimate the effects of tDCS.

A recent network meta-analysis compared third-generation ASMs, TMS, and tDCS against placebo in treating refractory epilepsy. They showed that NIBS was more effective than placebo, but the effectiveness of ASMs was superior to NIBS in reducing SF [42]. Our study demonstrated the updated evidence of tDCS regarding IEDs and SF compared to placebo. Reported adverse events after tDCS application were mild and not severe. Minor local itching, erythematous rash, tingling, and headache were reported and resolved spontaneously. Besides itching, Yang et al. 2019 reported seizure induction during the stimulation procedure [25]. This could be attributed to the non-linear effects of tDCS [43].

The studies indicate the varying efficacy of tDCS in reducing SF and IEDs. While Fregni et al. (2006) suggest that cathodal DC polarization can significantly reduce IEDs and shows a trend toward decreasing SF [33], Luo et al. (2021) noted a significant reduction in IEDs but not in SF [28]. This discrepancy highlights the need for further research to understand the mechanisms through which tDCS exerts its effects and to identify the optimal parameters for treatment.

The duration of tDCS effects is a topic of debate. Auvichayapat et al. (2016) found significant reductions in IEDs 48 h after c-tDCS treatment, with effects continuing to be observed at one-, two-, and three-week follow-ups. However, the reduction in IEDs was less noticeable at the four-week follow-up, indicating a decrease in efficacy over time while still providing some benefit [26], while Luo et al. (2021) found that a five-day consecutive c-tDCS treatment could reduce IEDs for at least four weeks [28]. Also, HD-tDCS produced an effect that remained significant over 12 weeks [37]. This variability in the duration of effects calls for more standardized protocols and long-term follow-up studies to ascertain the longevity of tDCS benefits.

There is ongoing discussion about the need for personalized stimulation protocols. Luo et al. (2021) suggest that personalized stimulation considering the site, intensity, and duration might yield better results as the epileptogenic origins of participants were heterogeneous [28]. This notion is supported by Rezakhani et al. (2022), who found significant inter-individual variability in response to tDCS [37].

Our study has several implications. The significant reduction in IEDs and SF suggests that tDCS could be integrated into clinical practice as a complementary treatment for patients with refractory epilepsy. These results provide substantial evidence of the efficacy of tDCS, potentially leading to its inclusion in epilepsy treatment guidelines. Clinicians might consider tDCS for patients who do not respond to traditional ASMs or are not suitable candidates for surgery. Many trials are being conducted to assess personalized and long-term effects of tDCS in refractory epilepsy [44–49].

The significant reductions in SF at various follow-up intervals indicate that tDCS may have lasting therapeutic effects, suggesting its potential use not only as an acute intervention but also as part of long-term maintenance therapy. Regularly scheduled tDCS sessions could help maintain seizure control over extended periods, improving patients’ quality of life and reducing the burden of frequent seizures.

Although our systematic review and meta-analysis highlight the potential of tDCS as an effective, non-invasive treatment for drug-resistant epilepsy, these implications emphasize the need for further research, personalized approaches, and potential integration into clinical practice to improve patient outcomes and quality of life.

### Limitations and future recommendations

This updated systematic review, and the first meta-analysis was conducted to investigate the efficacy and safety of tDCS in refractory epilepsy. Despite all included studies being RCTs comparing active tDCS versus sham tDCS, some limitations should be considered. The studies exhibit considerable heterogeneity in tDCS parameters such as electrode placement, current intensity, session duration, and treatment frequency. This variability complicates drawing definitive conclusions and limits the generalizability of our findings. Future research should standardize tDCS protocols to facilitate comparison across studies. Additionally, many studies have short follow-up periods, which do not adequately capture the long-term efficacy and safety of tDCS. Longer follow-up periods are needed to assess sustained effects and monitor delayed adverse effects.

The included studies did not report the type of ASMs used concurrently with tDCS. Therefore, we could not know if the use of concurrent medications depressed or enhanced tDCS effects. Furthermore, future studies should consider and assess the duration of seizures for patients before and after stimulation. It has been known that the longer the duration of the epilepsy, the more refractory it is [50, 51]. Thus, it is recommended to assess the duration of epilepsy of subjects since being diagnosed, to better understand the variability in response among patients based on their disease duration. This will be a critical factor in optimizing stimulation parameters tailored to each patient.

Another limitation is the small sample sizes in several studies, which limits the statistical power and the ability to detect significant differences between active and sham tDCS groups. The use of percent change from baseline in San Juan et al. 2022 was inefficient and not recommended to be used in RCTs. The statistical power of percent change from baseline is sensitive to any changes in the baseline distribution. Therefore, it may compromise the reliability, but, keeping into consideration that the percent change was reported for each individual, not for the whole group [23, 52]. Future studies should include larger sample sizes to improve the reliability and validity of the findings. Moreover, the included studies involve diverse patient populations with different types of epilepsy, varying ages, and different baseline seizure frequencies. This heterogeneity makes it difficult to identify which specific subgroups benefit most from tDCS. Future studies should focus on more homogeneous populations or conduct subgroup analyses to better understand the effects of tDCS in specific patient groups.

Our study could not assess the effects of tDCS on children and adults separately due to the limited number of focused studies, underscoring the need for more research targeting these groups. Our study highlights the need for focused studies, especially in children. Additionally, while some studies suggest mechanisms for the antiepileptic effects of tDCS, detailed mechanistic studies are lacking. Further research is needed to understand how tDCS modulates brain activity to reduce seizures. By addressing these limitations and following these recommendations, future research can advance the understanding and application of tDCS in treating drug-resistant epilepsy, ultimately improving patient outcomes and quality of life.

## Conclusion

The study favors active over sham tDCS in reducing monthly SF at four and eight weeks of follow-up and the number of IEDs at two, four, and eight weeks of follow-up. However, weekly SF showed no statistically significant results at four weeks of follow-up. The reported adverse events were mild and not severe. Taking into consideration the limitations of our study, future large RCTs employing consistent clear informed stimulation protocols are required to further promote tDCS therapy in the treatment of refractory epilepsy.

## Electronic supplementary material

Below is the link to the electronic supplementary material.

Supplementary Material 1

Supplementary Material 2

## Data Availability

Not applicable.
