# Supplementary material for: Efficacy and safety of transcranial direct current stimulation (tDCS) in treatment of refractory epilepsy: an updated systematic review and meta-analysis of randomized sham-controlled trials
Source: Neurol Sci. 2024 Nov 13;46(2):671–87. doi: 10.1007/s10072-024-07866-1 (PMC11772517; doi:10.1007/s10072-024-07866-1)
Supplement: Supplementary file 1 — Supplementary Material 1 [file 10072_2024_7866_MOESM1_ESM.pdf]

Efficacy and Safety of Transcranial Direct Current Stimulation (tDCS) in Treatment of Refractory Epilepsy: An Updated Systematic Review and Meta-Analysis of Randomized Sham-Controlled Trials.
"Neurological Sciences"

Yousef Hawas[1], Abdallah Abbas[2], Ibraheem M. Alkhawaldeh[3], Mohamed Abo Zeid[1], Mohammad Al Diab Al Azzawi[4], Hamza Khaled Alsalhi[5], Ahmed Negida[6,7]

1. Faculty of Medicine, Tanta University, Gharbeya, Egypt
2. Faculty of Medicine, Al-Azhar University, Damietta, Egypt
3. Faculty of Medicine, Mutah University, Al-Karak, Jordan
4. Faculty of Medicine, The National Ribat University, Khartoum, Sudan
5. Faculty of Medicine, The Hashemite University, Zarqa, Jordan
6. Department of Neurology, Virginia Commonwealth University, Richmond, Virginia, USA
7. Medical Research Group of Egypt, Negida Academy, Arlington, MA, USA.

Corresponding Author: Yousef Hawas. Email: yousef.ahmed.hawas7@gmail.com

The table demonstrates the excluded cross-over RCTs

Study ID	***Study design***	***Number of participants***	***Country***	***Number of centers***	***Refractory epilepsy type and etiology***	***tDCS electrodes***	***Cathode position***	***Anode position***	***Electrode size and current***	***Number and duration of sessions***	***Follow-up durations***	***Primary outcomes***	***Ameliorates***	***Adverse events***
**Assenza et al, 2017**	**Cross-over RCT**	**10 Adults**	**Italy**	**Single**	**Focal TLE (Varied etiology)**	**Conventional tDCS**	**EEG 10-20 (epileptogenic focus)**	**Contralateral Head**	**35 cm2, 1 mA**	**1 session for 20 min**	**1 week**	**EEG (19 electrodes), IEDs, SF**	**No change in IEDs, SF decreased by 71% in the first week of follow-up.**	**Skull Itching**
**Tekturk et al, 2016**	**Cross-over RCT**	**12 Adults**	**Turkey**	**Single**	**MTLE-HS**	**Conventional tDCS**	**EEG 10-20 (epileptogenic focus)**	**contralateral supraorbital**	**35 cm2, 2 mA**	**3 consecutive days for 30 minutes**	**2 months**	**SF**	**SF decreased by 84% up to the first month and was not sustained for the second month**	**Tingling**
**Varga et al, 2011**	**Cross-over RCT**	**5 Children**	**Denmark**	**Single**	**Focal (Varied etiology)**	**Conventional tDCS**	**"placed on the area of peak negativity (more restricted)"**	**"placed on the area of peak positivity (more widespread)"**	**25 cm2, 1 mA**	**2 sessions for 20 min**	**2 days**	**EEG (19 electrodes), IEDs**	**No change in IEDs during sleep**	**No adverse events**

TLE: *Temporal Lobe Epilepsy*, ***MTLE-HS****: Mesial temporal lobe epilepsy with hippocampal sclerosis*, ***NR:*** *Not reported*
