# Supplementary material for: Efficacy and safety of transcranial direct current stimulation (tDCS) in treatment of refractory epilepsy: an updated systematic review and meta-analysis of randomized sham-controlled trials
Source: Neurol Sci. 2024 Nov 13;46(2):671–87. doi: 10.1007/s10072-024-07866-1 (PMC11772517; doi:10.1007/s10072-024-07866-1)
Supplement: Supplementary file 2 — Supplementary Material 2 [file 10072_2024_7866_MOESM2_ESM.pdf]

Efficacy and Safety of Transcranial Direct Current Stimulation (tDCS) in Treatment of Refractory Epilepsy: An Updated Systematic Review and Meta-Analysis of Randomized Sham-Controlled Trials.

“Neurological Sciences”

Yousef Hawas[1], Abdallah Abbas[2], Ibraheem M. Alkhawaldeh[3], Mohamed Abo Zeid[1], Mohammad Al Diab Al Azzawi[4], Hamza Khaled Alsalhi[5], Ahmed Negida[6,7]

1. Faculty of Medicine, Tanta University, Gharbeya, Egypt
2. Faculty of Medicine, Al-Azhar University, Damietta, Egypt
3. Faculty of Medicine, Mutah University, Al-Karak, Jordan
4. Faculty of Medicine, The National Ribat University, Khartoum, Sudan
5. Faculty of Medicine, The Hashemite University, Zarqa, Jordan
6. Department of Neurology, Virginia Commonwealth University, Richmond, Virginia, USA
7. Medical Research Group of Egypt, Negida Academy, Arlington, MA, USA.

Corresponding Author: Yousef Hawas. Email: yousef.ahmed.hawas7@gmail.com

In this supplement, we have made further analysis for the seizure frequency (SF) outcome. We used standardized mean difference (SMD) to reduce the differences in the measurement units of SF between the included studies. SMD expresses the size of the mean difference in standard deviation units. However, it is considered less efficient than the raw mean difference when directly comparing outcomes measured on the same scale. the SMD presents results in statistical units, whereas the mean difference presents results in clinical units [1]. This supplementary provides a glimpse of the tDCS effect in standard deviation units.

We used SMD with 95% confidence intervals and adopted the random effect model. The random effect model assumes that the included studies are random samples from the population. It calculates the effect estimate with larger SE and wider CI. In addition, studies as Yang et al. 2019 and San Juan et al. 2016, included three arms; two intervention groups of active tDCS with different stimulation parameters, and one control group with sham tDCS. We combined the reported outcome data of the intervention groups together based on the following formula [2] to avoid duplicating the same control group which would affect the overall effect estimate.

Fig.1 showed a significant reduction in SF in the active tDCS group compared to sham tDCS at week 2 (SMD = -0.88, 95% CI = [-1.64 to -0.12], P = 0.02) and week 4 of follow-ups (SMD = -0.43, 95% CI = [-0.8 to -0.05], P = 0.03). However, at week 8, there was no significant difference between the active and sham groups. The overall SMD between active and sham tDCS favored active tDCS (Pooled SMD = -0.49, 95% CI = [-0.76 to -0.22], P = 0.0004). Pooled studies were not homogenous (P = 0.1), with no significant heterogeneity (I^2 = 35%).

In **Fig. 2,** there was no statistically significant difference between active and sham tDCS groups for those below or above 18 years of age at 4 weeks of follow-up. Subgroup analysis based on stimulation intensity was found to be statistically insignificant for both those who used 1mA and 2mA at 4 weeks of follow-up **(Fig. 3)**.

We believe that the heterogeneity that presents at 2, and 4 weeks of follow-up, in those who were above 18 years of age, and among those who used 2mA stimulation is because Rezkhani et al. 2022 used HD-tDCS while all studies used conventional (one cathode one anode) tDCS. Rezkhani et al. 2022 used HD-tDCS (smaller five cathodal electrodes and one anode), this focused delivery enables a higher intensity of stimulation to reach the cortex, resulting in a powerful effect. Additionally, differences in stimulation parameters, such as the number and duration of sessions, may also contribute to the observed heterogeneity. Rezkhani et al. 2022 applied HD-tDCS for 10 sessions each for 30 minutes, which is considered longer sessions compared to the included studies, except for Yang et al 2019 who applied cathodal tDCS for 14 sessions: one group for 20 minutes and another for 40 minutes with 20 minutes interval (20-20-20).

These variations denote that clear informed stimulation protocols are required to further promote tDCS therapy in the treatment of refractory epilepsy.

Study or Subgroup	Active tDCS Mean	Active tDCS SD	Active tDCS Total	Sham tDCS Mean	Sham tDCS SD	Sham tDCS Total	Weight	Std. mean difference IV, Random, 95% CI
**2.2.1 at 2 weeks**								
2. Auvichayabat 2016	-65.47	53.43	15	-25.94	63.25	7	6.2%	-0.67 [-1.60 , 0.25]
2. Rezakhani 2022	-6.3	2.36	10	-0.7	3.37	10	4.9%	-1.84 [-2.93 , -0.76]
2. Yang 2019	-2.24	5.458	49	0.856	8.875	21	12.5%	-0.46 [-0.98 , 0.06]
**Subtotal**			**74**			**38**	**23.6%**	**-0.88 [-1.64 , -0.12]**
Test for overall effect: Z = 2.28 (P = 0.02)								
Heterogeneity: Tau² = 0.27; Chi² = 5.10, df = 2 (P = 0.08); I² = 61%								
**2.2.2 at 4 weeks**								
4. Ashrafzadeh 2023	0.4	26.7	9	-0.22	14.75	9	6.2%	0.03 [-0.90 , 0.95]
4. Auvichayabat 2016	-45.14	53.51	15	-13.7	60.52	7	6.3%	-0.54 [-1.46 , 0.37]
4. Fregni 2006	-3.7	6.89	10	0.8	5.66	9	6.2%	-0.68 [-1.61 , 0.25]
4. Rezakhani 2022	-4.5	2.37	10	0.1	2.72	10	5.1%	-1.73 [-2.79 , -0.67]
4. San juan 2016	-3.75	6.23	20	-3.75	9.646	8	7.4%	0.00 [-0.82 , 0.82]
4. Yang 2019	-1.53	5.627	49	-0.132	8.6	21	12.6%	-0.21 [-0.72 , 0.30]
4. Zoghi 2016	-35.82	78.03	16	-7.28	34.34	7	6.5%	-0.40 [-1.30 , 0.50]
**Subtotal**			**129**			**71**	**50.2%**	**-0.43 [-0.80 , -0.05]**
Test for overall effect: Z = 2.20 (P = 0.03)								
Heterogeneity: Tau² = 0.08; Chi² = 8.71, df = 6 (P = 0.19); I² = 31%								
**2.2.3 at 8 weeks**								
8. Rezakhani 2022	-2.5	2.68	10	0.4	3.42	10	6.2%	-0.90 [-1.83 , 0.03]
8. San juan 2016	-4.4	5.994	20	-2.75	9.981	8	7.3%	-0.22 [-1.04 , 0.60]
8. Yang 2019	-1.52	6.287	49	-0.889	8.426	21	12.6%	-0.09 [-0.60 , 0.42]
**Subtotal**			**79**			**39**	**26.1%**	**-0.28 [-0.72 , 0.15]**
Test for overall effect: Z = 1.28 (P = 0.20)								
Heterogeneity: Tau² = 0.02; Chi² = 2.28, df = 2 (P = 0.32); I² = 12%								
**Total**			**282**			**148**	**100.0%**	**-0.49 [-0.76 , -0.22]**
Test for overall effect: Z = 3.53 (P = 0.0004)								
Test for subgroup differences: Chi² = 1.82, df = 2 (P = 0.40), I² = 0%								
Heterogeneity: Tau² = 0.08; Chi² = 18.56, df = 12 (P = 0.10); I² = 35%								

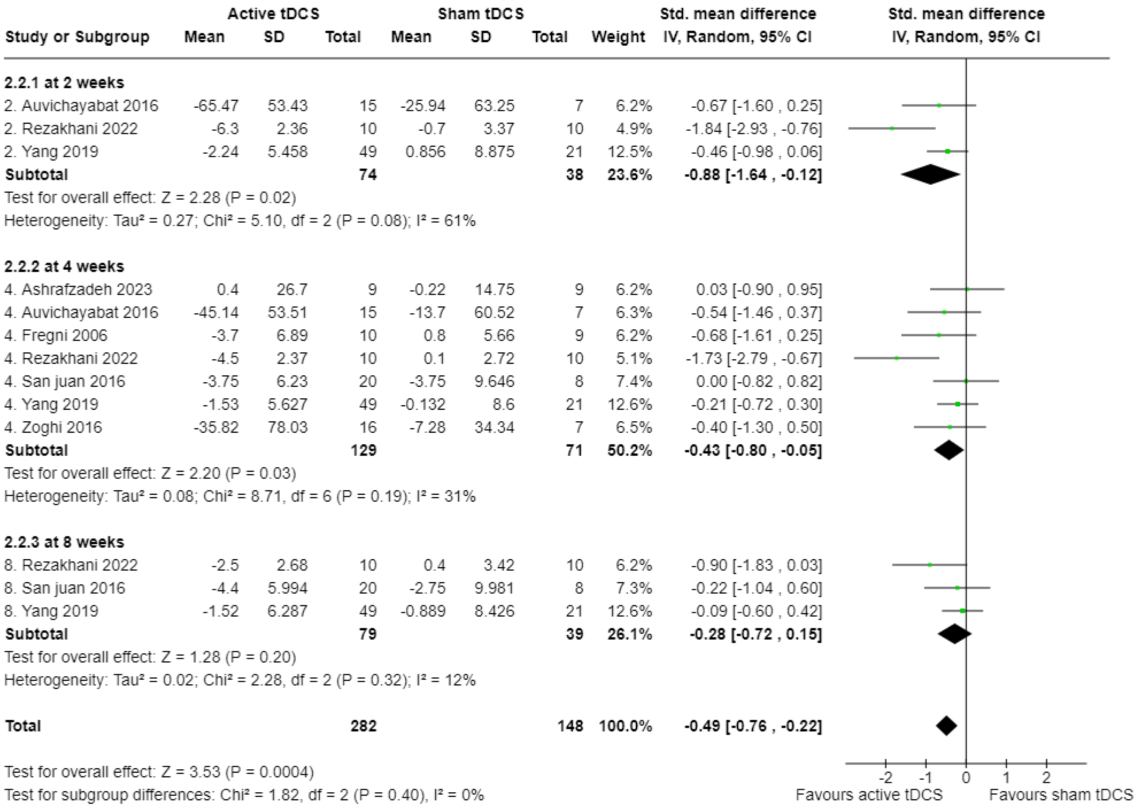

Fig.1 Seizure frequency sub-grouped based on follow-up periods.

Study or Subgroup	Active tDCS Mean	Active tDCS SD	Active tDCS Total	Sham tDCS Mean	Sham tDCS SD	Sham tDCS Total	Weight	Std. mean difference IV, Random, 95% CI
**2.3.1 Adults (≥ 18 years old)**								
4. Rezakhani 2022	-4.5	2.37	10	0.1	2.72	10	12.0%	-1.73 [-2.79 , -0.67]
4. San juan 2016	-3.75	6.23	20	-3.75	9.646	8	16.9%	0.00 [-0.82 , 0.82]
4. Yang 2019	-1.53	5.627	49	-0.132	8.6	21	26.8%	-0.21 [-0.72 , 0.30]
4. Zoghi 2016	-35.82	78.03	16	-7.28	34.34	7	15.1%	-0.40 [-1.30 , 0.50]
**Subtotal**			**95**			**46**	**70.8%**	**-0.49 [-1.12 , 0.13]**
Test for overall effect: Z = 1.54 (P = 0.12)								
Heterogeneity: Tau² = 0.24; Chi² = 7.45, df = 3 (P = 0.06); I² = 60%								
**2.3.2 Children (< 18 years old)**								
4. Ashrafzadeh 2023	0.4	26.7	9	-0.22	14.75	9	14.5%	0.03 [-0.90 , 0.95]
4. Auvichayabat 2016	-45.14	53.51	15	-13.7	60.52	7	14.7%	-0.54 [-1.46 , 0.37]
**Subtotal**			**24**			**16**	**29.2%**	**-0.26 [-0.91 , 0.39]**
Test for overall effect: Z = 0.79 (P = 0.43)								
Heterogeneity: Tau² = 0.00; Chi² = 0.74, df = 1 (P = 0.39); I² = 0%								
**Total**			**119**			**62**	**100.0%**	**-0.40 [-0.83 , 0.03]**
Test for overall effect: Z = 1.81 (P = 0.07)								
Test for subgroup differences: Chi² = 0.25, df = 1 (P = 0.61), I² = 0%								
Heterogeneity: Tau² = 0.11; Chi² = 8.29, df = 5 (P = 0.14); I² = 40%								

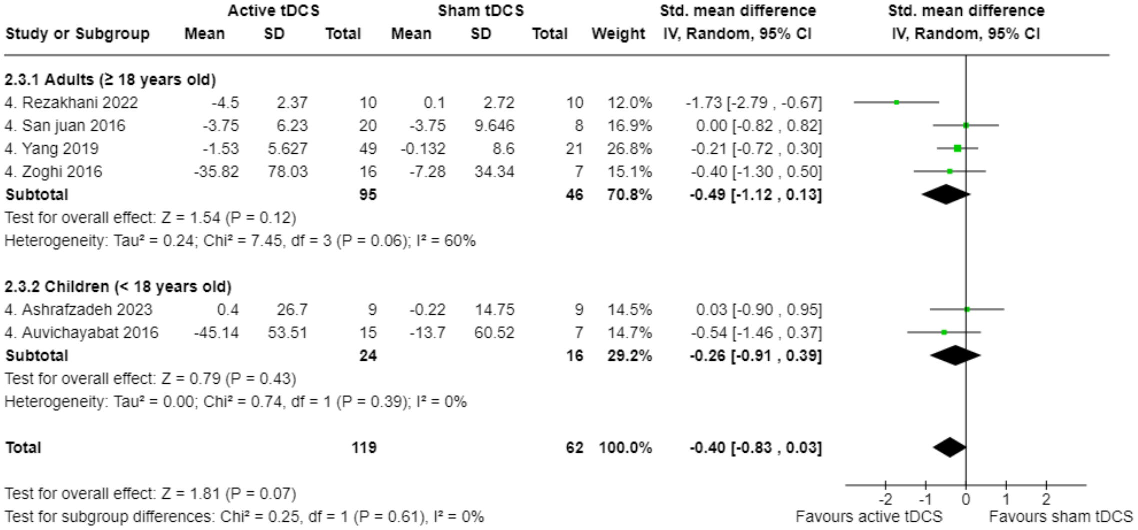

Fig. 2 Seizure frequency sub-grouped based on age at four weeks of follow-up.

Study or Subgroup	Active tDCS Mean	Active tDCS SD	Active tDCS Total	Sham tDCS Mean	Sham tDCS SD	Sham tDCS Total	Weight	Std. mean difference IV, Random, 95% CI
**2.1.1 1mA**								
4. Ashrafzadeh 2023	0.4	26.7	9	-0.22	14.75	9	12.4%	0.03 [-0.90 , 0.95]
4. Fregni 2006	-3.7	6.89	10	0.8	5.66	9	12.2%	-0.68 [-1.61 , 0.25]
4. Zoghi 2016	-35.82	78.03	16	-7.28	34.34	7	12.9%	-0.40 [-1.30 , 0.50]
**Subtotal**			**35**			**25**	**37.5%**	**-0.35 [-0.88 , 0.18]**
Test for overall effect: Z = 1.29 (P = 0.20)								
Heterogeneity: Tau² = 0.00; Chi² = 1.13, df = 2 (P = 0.57); I² = 0%								
**2.1.2 2mA**								
4. Auvichayabat 2016	-45.14	53.51	15	-13.7	60.52	7	12.6%	-0.54 [-1.46 , 0.37]
4. Rezakhani 2022	-4.5	2.37	10	0.1	2.72	10	10.0%	-1.73 [-2.79 , -0.67]
4. San juan 2016	-3.75	6.23	20	-3.75	9.646	8	14.7%	0.00 [-0.82 , 0.82]
4. Yang 2019	-1.53	5.627	49	-0.132	8.6	21	25.2%	-0.21 [-0.72 , 0.30]
**Subtotal**			**94**			**46**	**62.5%**	**-0.53 [-1.16 , 0.11]**
Test for overall effect: Z = 1.63 (P = 0.10)								
Heterogeneity: Tau² = 0.25; Chi² = 7.56, df = 3 (P = 0.06); I² = 60%								
**Total**			**129**			**71**	**100.0%**	**-0.43 [-0.80 , -0.05]**
Test for overall effect: Z = 2.20 (P = 0.03)								
Test for subgroup differences: Chi² = 0.18, df = 1 (P = 0.67), I² = 0%								
Heterogeneity: Tau² = 0.08; Chi² = 8.71, df = 6 (P = 0.19); I² = 31%								

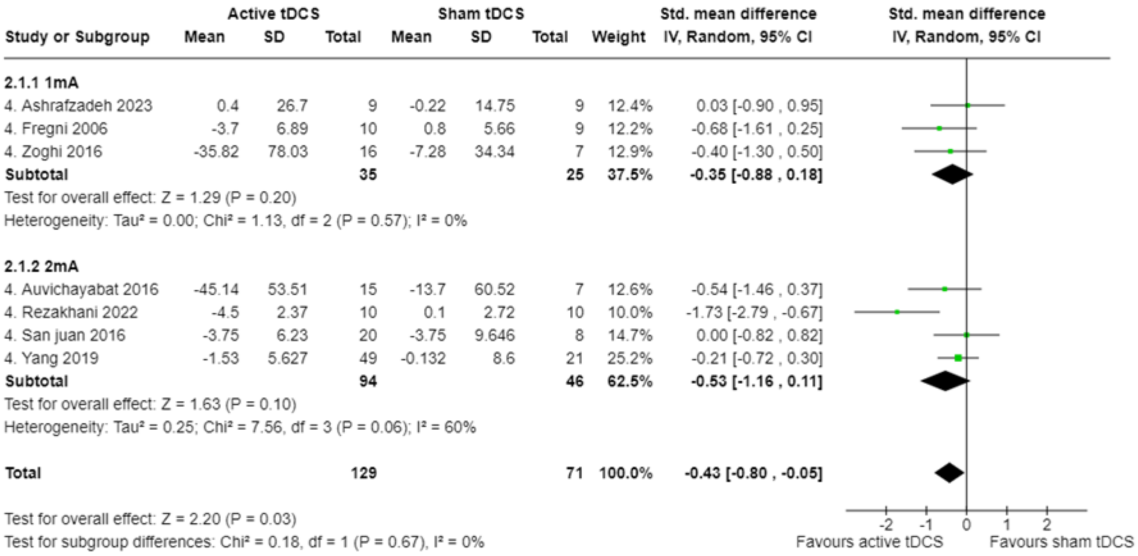

Fig. 3 Seizure frequency sub-grouped based on stimulation intensity at four weeks of follow-up.

References

1. Andrade C (2020) Mean Difference, Standardized Mean Difference (SMD), and Their Use in Meta-Analysis: As Simple as It Gets. J Clin Psychiatry 81:11349. https://doi.org/10.4088/JCP.20f13681

2. Higgins JP, Li T, Deeks JJ Chapter 6.5.2.10: Choosing effect measures and computing estimates of effect; continuous outcome data; Data extraction for continuous outcome; Combining groups. https://training.cochrane.org/handbook/current/chapter-06#section-6-5-2-10. Accessed 27 Sep 2024
